# Preparation of Mn/Zn@PG Catalyst for Catalytic Oxidation Treatment of Coal Chemical Wastewater

**DOI:** 10.3390/ijerph191710812

**Published:** 2022-08-30

**Authors:** Wenquan Sun, Shuqian Xie, Yongjun Sun, Xiangtao Qiu, Jun Zhou

**Affiliations:** College of Urban Construction, Nanjing Tech University, Nanjing 211816, China

**Keywords:** ozone catalyst, attapulgite, coal chemical wastewater, ozone oxidation, comprehensive evaluation and analysis

## Abstract

In this study, Mn/Zn@palygorskite (PG) catalysts with developed pores and good salt tolerance were prepared and applied to the treatment of coal chemical wastewater. A doping ratio of metal elements, calcination temperature, and calcination time was used to optimize the preparation conditions and determine the optimal preparation conditions of the Mn/Zn@PG catalysts. The catalysts, obtained under various preparation conditions, were characterized and analyzed by XRD, SEM, EDS, BET, XRF, XPS, and other techniques. Results showed that the Zn and Mn elements in the Mn/Zn@PG catalyst existed as ZnO and MnO_2_, respectively. The optimal working conditions of the Mn/Zn@PG catalyst for catalytic oxidation treatment of coal chemical wastewater, obtained through the optimization of working conditions, are the following: reaction time 60 min, wastewater pH = 9.28, ozone ventilation rate 0.2 L/min, catalyst filling ratio 20%. The height-to-diameter ratio of the tower was 6:1. The abrasion resistance and catalytic performance of the Mn/Zn@PG catalyst after repeated use were investigated, and the mechanism of the loss of active components of the Mn/Zn@PG catalyst was explored. The coal chemical wastewater, before and after treatment, was analyzed by UV–vis spectroscopy and 3D fluorescence spectroscopy. The hierarchical–principal component comprehensive evaluation system (AHP–PCA) was established to evaluate the catalytic ozonation process of coal chemical wastewater, so that the overall evaluation of the process performance can be achieved.

## 1. Introduction

With rapid economic development, human demand for resources has increased significantly, and the contradiction between energy supply and demand has become increasingly prominent. Although renewable and clean energy shows great potential for development, it cannot meet the current social needs, due to cost and technology constraints. The development of new coal chemical industries have realized the transformation of coal resources into clean and renewable energy, but the pollution problem has not been effectively dealt with [1].

Coal chemical wastewater contains many kinds of toxic, harmful, and refractory pollutants, and its pollutant concentration is high [2]. Treating these pollutants efficiently has become a major industrial difficulty, both locally and internationally. Coal chemical wastewater can be divided into salt-containing wastewater and organic wastewater, according to the salt content. The salt-containing wastewater mainly comes from the gas washing wastewater in the coal chemical production process, drainage of the circulating water system, demineralized water system, and concentration of the water reuse system [3]. Organic wastewater mainly includes coal gasification wastewater, comprehensive chemical wastewater, and desulfurization wastewater. Coal chemical wastewater has the characteristics of high concentration, high toxicity and carcinogenicity, complex composition, and a large amount of water, including a large number of phenols, aromatic hydrocarbons, long-chain alkanes, nitrogen-containing heterocycles, ammonia nitrogen, cyanide, and other substances [4]. If coal chemical wastewater is discharged into bodies of water without proper treatment, it will cause serious harm to people, animals, and crops around the water area. Therefore, developing an effective treatment process to improve the biodegradability of coal chemical wastewater is significant in practice.

Advanced oxidation processes are widely applied to degrade harmful organic pollutants in industrial wastewater [5]. Among these processes, ozone oxidation technology has received extensive attention, due to its high oxidation efficiency, fast reaction speed, and absence of secondary pollutants [6]. However, the solubility and mass transfer efficiency of this technology are very poor, resulting in low utilization in the reaction system [7,8]. To date, several types of heterogeneous catalysts have been used to catalyze ozone oxidation reactions, such as manganese oxides, iron oxides/hydroxyl oxides, aluminum and bimetallic/polymetallic oxides, carbon-based materials, and metal/metal oxides supported on the carrier. Due to the addition of various types of catalysts, the decomposition of ozone becomes more efficient, which can produce more highly active free radicals and promote the oxidation of organic matter. At the same time, heterogeneous catalytic oxidation technology has the advantages of easy catalyst recovery, low water treatment cost, and high catalyst activity [4,9,10]. However, the catalytic ozonation reaction mechanism is complex, poor durability of individual catalysts makes its cost high, and catalyst may be deactivated, due to high salinity during the reaction process. These problems limit the application of ozone catalytic oxidation technology in industrial wastewater treatment. Therefore, this study aimed to prepare an efficient and stable multi-component metal catalyst for the treatment of high-salinity coal chemical wastewater, as well as to investigate its catalytic degradation performance and salt resistance stability in wastewater treatment. The objective is to improve the ozone catalysis degradation efficiency of the oxidative treatment of coal chemical wastewater and provide a reference for related research.

In this study, Mn/Zn@PG catalysts with developed pores and good salt tolerance were prepared. The catalysts under different conditions were characterized by XRD, SEM, EDS, BET, XRF, and XPS. The effects of the height diameter ratio of the reaction tower, gas flow rate, reaction time, catalyst filling rate, pH value, and ozone dosage on the catalyst performance were investigated. Through single-factor optimization, the optimal process conditions for the treatment of coal chemical wastewater by ozone catalytic oxidation were determined. The types and changes of organic compounds in coal chemical wastewater, before and after treatment, were analyzed by UV absorption peaks and 3D fluorescence spectra, and the reaction mechanism of ozone catalytic oxidation system was explored by adding free radical quenchers. By establishing a comprehensive evaluation system of hierarchy–principal component, the ozone catalytic oxidation treatment test of coal chemical wastewater was evaluated to obtain the best working conditions.

## 2. Materials and Methods

### 2.1. Experimental Materials

Sodium silicate, manganese nitrate, zinc nitrate, copper nitrate, cerium nitrate, and bismuth nitrate were of analytical grade and purchased from Sinopharm Chemical Reagent Co., Ltd. Soluble starch (Shanghai, China). Nessle’s reagent, potassium dichromate, silver sulfate, and mercury sulfate were of analytical grade and purchased from Nanjing Chemical Reagent Co., Ltd. (Nanjing, China) Palygorskite was 400 mesh, purchased from Changzhou Dingbang Mineral Products Co., Ltd. (Changzhou, China) Coal chemical wastewater was taken from a coal chemical enterprise in Yunnan and used in the experiment. The water sample was gray-brown, dark in color, and turbid. The water quality was shown in Table 1.

### 2.2. Preparation of Mn/Zn@PG Catalyst

A quantitative amount of palygorskite powder, pore-forming agent, and binder were taken and stirred evenly in a beaker for later use. A quantitative amount of nitrate solution of Cu, Mn, Ce, Bi, and Zn was weighed, diluted to 100 mL in a volumetric flask, shaken evenly, and placed in a beaker as a precursor solution for later use. The precursor solution in the beaker was poured into the uniformly mixed palygorskite powder, pore-forming agent, and binder. The container was shaken vigorously to mix the contents evenly and then placed in an oven to dry at 80 °C. The dried palygorskite was taken out to make a spherical catalyst, and the diameter of the catalyst sphere was controlled within 5–6 mm. The dried catalyst was placed in a crucible and calcined at a certain temperature in a muffle furnace. After the calcination was completed, the calcined catalyst was taken out, exposed to the air for natural cooling, and finally packaged for use.

### 2.3. Characterization of Mn/Zn@PG Catalysts

The surface micromorphology of the Mn/Zn@PG catalyst Mn/Zn@PG catalyst was characterized by SEM (Zeiss Merlin, Zeiss, Jena, Germany). The crystal morphology of the metal in the Mn/Zn@PG catalyst was characterized by XRD (D8 Advance, Burker, Germany). The contents of metal elements and metal oxides in the Mn/Zn@PG catalysts were characterized by XRF (Axios Pw4400, PANalytical, Almelo, The Netherlands). Compressive strength analysis (AG-X 50 kN, Shimadzu Corporation, Tokyo, Japan) was used to characterize the abrasion resistance and compressive resistance of Mn/Zn@PG ozone catalysts. The content of metal elements in the catalysts was analyzed by energy dispersive X-ray spectroscopy (EDS) (Kratos AXIS Ultra, Shimadzu Corporation, Japan). The metal element composition and valence state of the catalyst were characterized by XPS (250xi, Perkin Elmer, Waltham, MA, USA). BET analysis (ASAP-2020, Micromeritics, Norcross, GA, USA) was used to characterize the pore volume, pore size, and adsorption performance of the catalysts.

### 2.4. Ozone Catalytic Oxidation Experiment

The catalytic reaction device consisted of an oxygen cylinder, ozone generator (CF-G-3-10G, Qingdao Guolin, Qingdao, China), gas flow meter, reaction tower, and tail gas absorption device. Before the start of the experiment, the oxygen source was opened to pre-blow the ozone generator for 10 min. After the pre-blow, a quantitative amount of the prepared catalyst was added, and 400 mL of coal chemical wastewater was placed in the reaction tower. The cooling water was switched on, and the gas flow and inlet pressure were adjusted. Then, the ozone generator was turned on, and the oxygen generated ozone through high-voltage discharge inside the ozone generator. Water samples were taken every 10 min after the experiment started. By adjusting the gas flow meter of the ozone generator, we controlled the output of ozone, reserved a water outlet on the side wall of the reactor, and took samples regularly during the catalytic oxidation reaction to measure various indicators. The exhaust gas generated in the experiment was treated by the exhaust gas absorption device (20% KI solution) and discharged. After the experiment, the ozone generator and cooling water return device were closed, and high-purity oxygen was continuously introduced into the ozone generator for 10 min to prevent the ozone generator. When the dust pollution was over, the main valve of the oxygen cylinder was closed.

### 2.5. Water Quality Analysis Method

In this experiment, iodometric method was used to detect the concentration of ozone in the gas phase [11]. In this experiment, the indigo method was used to measure the ozone concentration in the liquid phase. To explore the changes in the types of organic substances in the coal chemical wastewater treatment process, the coal chemical wastewater, before and after treatment, was analyzed by UV-full wavelength scanning. The detection wavelength was 190–1100 nm, and the scanning wavelength interval was 0.1 nm. Using a 3D fluorescence spectrometer to analyze the wastewater before and after treatment, more information on the types and changes of organic matter in the water can be obtained. The detection range of excitation wavelength (Ex) was 250–500 nm, and the detection range of emission wavelength (Em) was 300–600 nm. The leaching concentration of metal ions in wastewater, before and after treatment, was analyzed by atomic absorption spectrometry, and the stability of the catalyst was investigated.

### 2.6. Hierarchical–Principal Component Comprehensive Evaluation Model Analysis

This study established three levels: target, criterion, and index. The comprehensive evaluation system took the three aspects of environmental benefit, cost consumption, and energy consumption as the criterion layers and selected seven criteria: COD removal rate, total phenol removal rate, chromaticity removal rate, height–diameter ratio, catalyst filling ratio, ozone ventilation, and reaction pH. The evaluation index was the index layer. According to the selected specific evaluation indexes, a comprehensive evaluation index system of Mn/Zn@PG catalyst for catalytic ozonation of coal chemical wastewater was constructed. Its structure and operation process are shown in TEXT S1.

## 3. Results and Discussion

### 3.1. Catalyst Preparation and Optimization

As shown in Figure S2, with the increase of Mn element ratio, the removal of COD and chromaticity of wastewater first increased and then decreased. When Zn:Mn = 1:2, the removal rates of COD and chroma reached the highest levels, which were 55.8% and 93.0%, respectively. Continuing to increase the ratio of Mn element, when Zn:Mn = 1:3, the removal rates of COD and chroma decreased to 49.4% and 91.2%, respectively. This phenomenon is attributed to the fact that proper catalyst composition is significant to the performance of catalyst. Insufficient metal oxide loaded on the supporter leads to the lack of active sites, while excess metal oxide might generate clusters on the surface of catalyst, causing the decline of catalytic activity. Figure S3 shows that the optimal calcination temperature of the Mn/Zn@PG catalyst is 400 °C; at this temperature, the optimal removal rates of COD and chroma are 55.8% and 93.0%, respectively. Figure S4 shows that, when the calcination time reaches 4 h, the optimal removal rates of COD and chroma are 55.8% and 91.6%, respectively, and the catalytic activity of the Mn/Zn@PG catalyst is most optimal at this time.

### 3.2. Characterization of Catalysts

**Figure 1:**
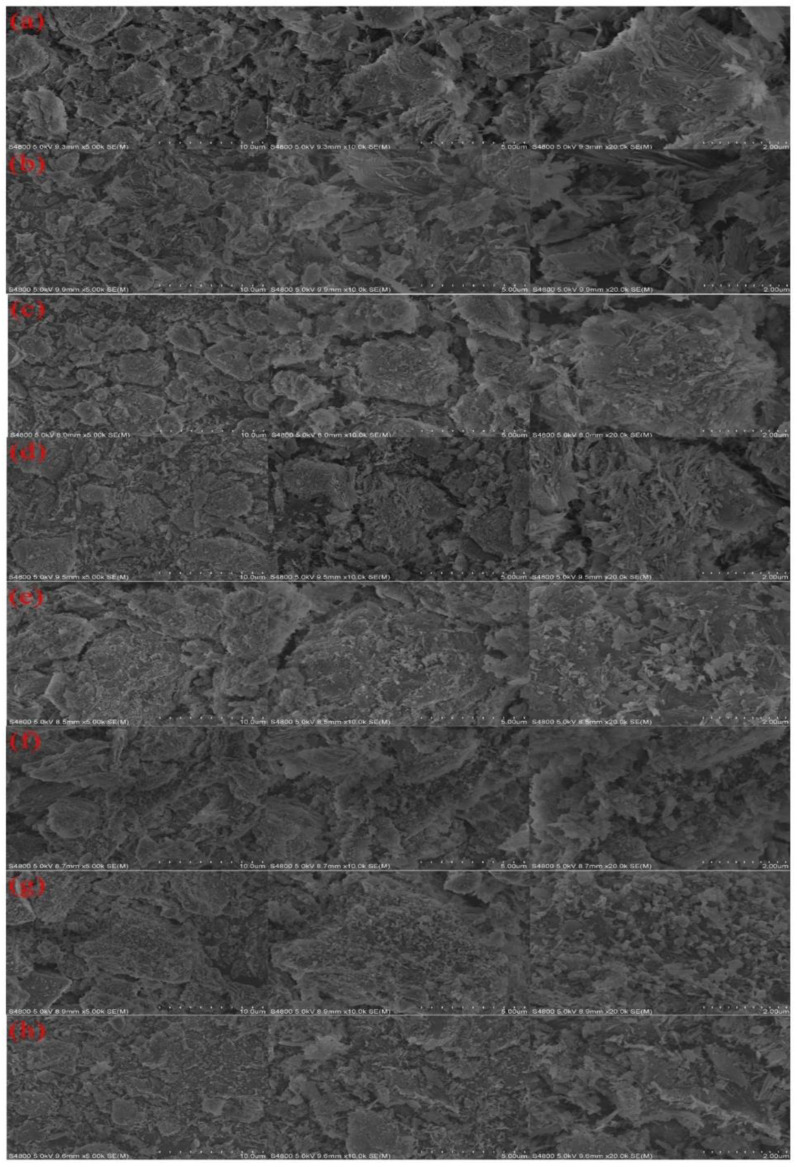
SEM of Mn/Zn@PG catalyst: (**a**) blank; (**b**) 100 °C; (**c**) 250 °C; (**d**) 400 °C; (**e**) 550 °C; (**f**) 700 °C; (**g**) 850 °C; (**h**) Mn/Zn@PG after utilization 20 times.

According to Figure S5 and PDF, cards such as SiO_2_ (PDF#99-0088), MgO_2_ (PDF#19-0771), Al_2_O_3_ (PDF#99-0036), and palygorskite itself contains a large amount of elements, such as Si, Mg, and Al. Compared with the diffraction peaks of the blank catalyst, other samples have obvious peaks at 20.211°, 21.551°, 31.766°, 36.251°, 36.357°, 56.591°, 67.942°, and so on, which correspond to the MnO_2_ (100), (110), (021), and (050) planes (PDF #89-2804) [12]. After high temperature calcination, Mn and Zn elements exist in the form of MnO_2_ and ZnO, respectively, in the catalyst; the diffraction peaks at 2θ = 31.766°, 36.251°, 56.591°, 67.942°, and others correspond to (100) and (101) of ZnO, respectively, (110) and (112) (PDF #99-0111) [13]. When the calcination temperature increased from 100 °C to 400 °C, the characteristic peak intensities of MnO_2_ and ZnO gradually increased. When the temperature was between 550 and 850 °C, the characteristic peak intensity of the MnO_2_ and ZnO crystals does not increase significantly with the increase of temperature, and the diffraction peak intensity at 2θ = 36.357° and so on decreases. With the increase in the number of metal crystals supported on the catalyst, the crystals cover one another. Figure 1 shows that palygorskite has a rod-like fiber structure with abundant pores. When the calcination temperature was 400 °C, many crystal particles were observed on the surface of the catalyst, and the catalyst had the best performance at this time (shown in Figure S3). When the calcination temperature continues to increase, the catalyst surface is loaded with a large number of larger crystal particles, so that too many crystal particles cover the surface of the carrier and melting occurs, resulting in a serious reduction of the specific surface area and pore structure of the catalyst, as well as the reduction of the catalyst in the active site [14]. When the catalyst was used 20 times, its pores were blocked by a large amount of organic matter, which reduced the catalytic performance of the Mn/Zn@PG catalyst [15].

Table 2 shows that the mass fractions of ZnO and MnO_2_ were 5.47 and 10.65 wt%, respectively, which indicates that Zn and Mn elements were successfully loaded into the catalyst. The content of most metal elements in the Mn/Zn@PG catalyst after 20 uses decreased; the mass fractions of ZnO and MnO_2_ were 5.3694 and 10.3719 wt%, respectively, and still had a high content of active components. Table S13 shows that the specific surface area, average pore volume, and average pore size of the Mn/Zn@PG catalyst were smaller than those of the blank catalyst, which may be due to a large amount of metal oxides loaded on the surface and inside the pores of the carrier, after being modified by high-temperature calcination. Figure S6d shows that the pore size of the blank support was mainly distributed around 50 nm, including the mesopores and macropores, while the main pore size of the Mn/Zn@PG catalyst was mainly distributed at 25 nm. As shown in Figure S6a–c, the three adsorption and desorption isotherms all conformed to the type IV isotherm, with a H3-type hysteresis loop, and mesopores with a wedge-shaped structure exist [16]. An obvious mesoporous hysteresis loop appeared at 0.2 < P/Po < 0.8, and an obvious macroporous hysteresis loop appeared at 0.8 > P/Po, which indicated that the main adsorption modes of Mn/Zn@PG catalysts were mesoporous and macroporous adsorption [17].

**Figure 2:**
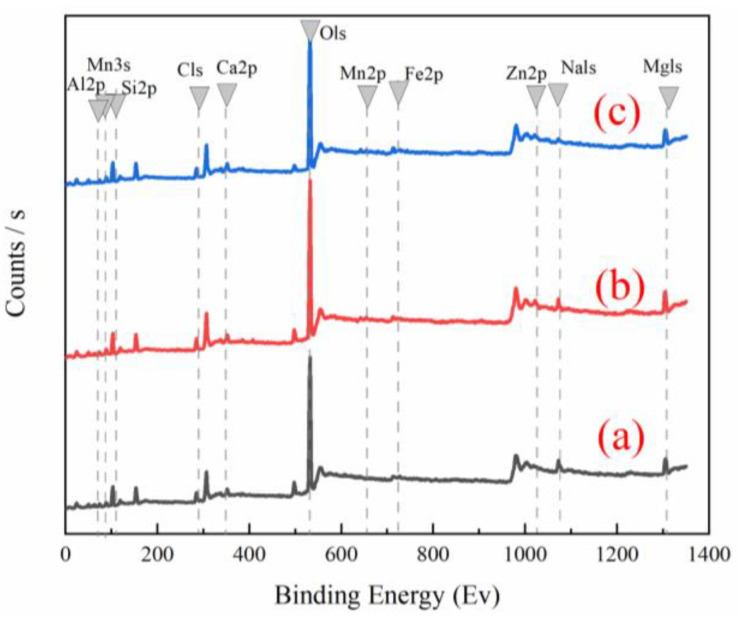
XPS characterization: (**a**) blank; (**b**) Mn/Zn@PG; (**c**) Mn/Zn@PG after utilization 20 times.

Figure S7 shows that the weight percentages of Mn and Zn elements in palygorskite were 0.11 and 0.35 wt%, respectively, while the weight percentages of Mn and Zn elements in the Mn/Zn@PG catalyst were 5.65 and 3.24 wt%, respectively. This result indicates that Mn and Zn elements have been successfully loaded into the catalyst. This is consistent with the result of EDS in Table 3. The weight percentages of Mn and Zn elements in the Mn/Zn@PG catalyst used 20 times were 1.86 and 1.51 wt%, respectively, which may be due to the loss of the elements supported on the catalyst surface, due to collision and friction between catalysts during use. Figure S8 and Figure 2 show that Zn2p has two peaks at the electron potential energy of 1044.95 and 1021.70 eV, and the spin-orbit splitting of 23.25 eV appeared in the Zn2p core energy level spectrum, indicating that the atomic valence state of Zn element in the Mn/Zn@PG catalyst was +2 [18]. Mn2p has two peaks at the electron potential energies of 653.30 and 642.20 eV, while the spin-orbit splitting of 11.1 eV appeared in the Mn2p core energy spectrum, indicating that the atomic valence of Mn element in the Mn/Zn@PG catalyst was +4 [19].

### 3.3. Optimization of Working Conditions for Catalyst Degradation of Coal Chemical Wastewater

**Figure 3:**
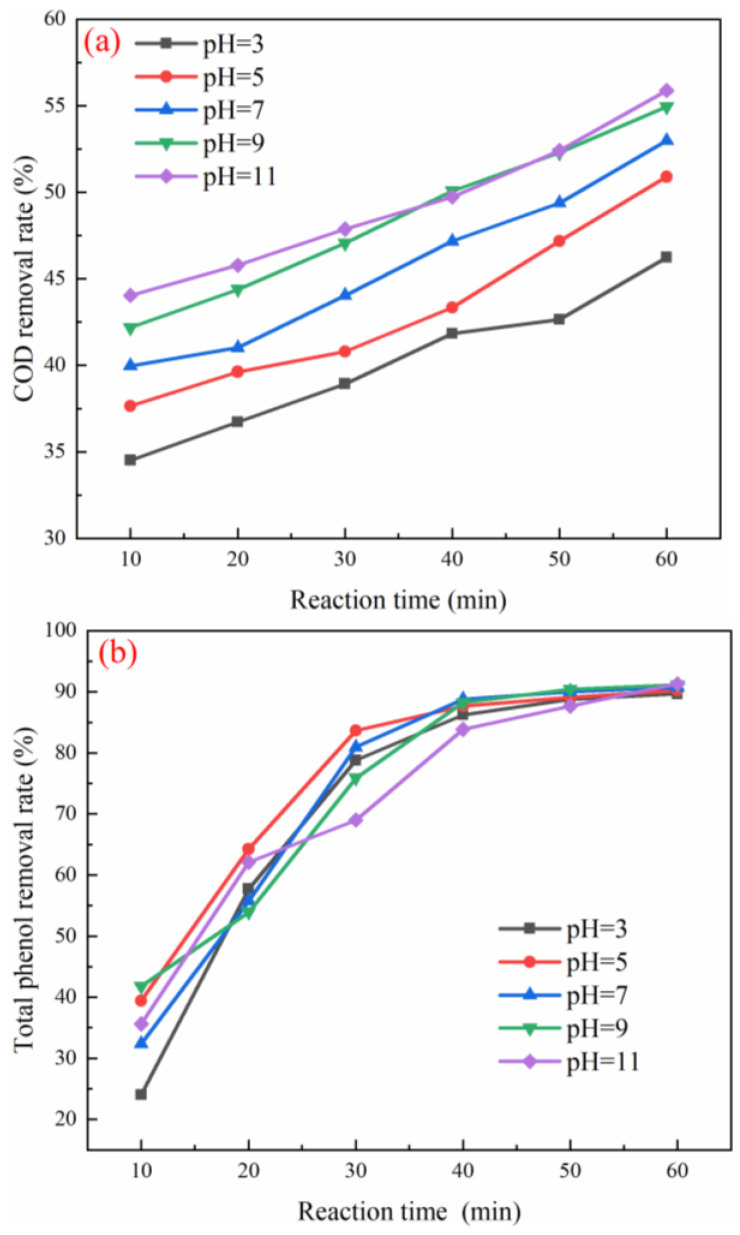

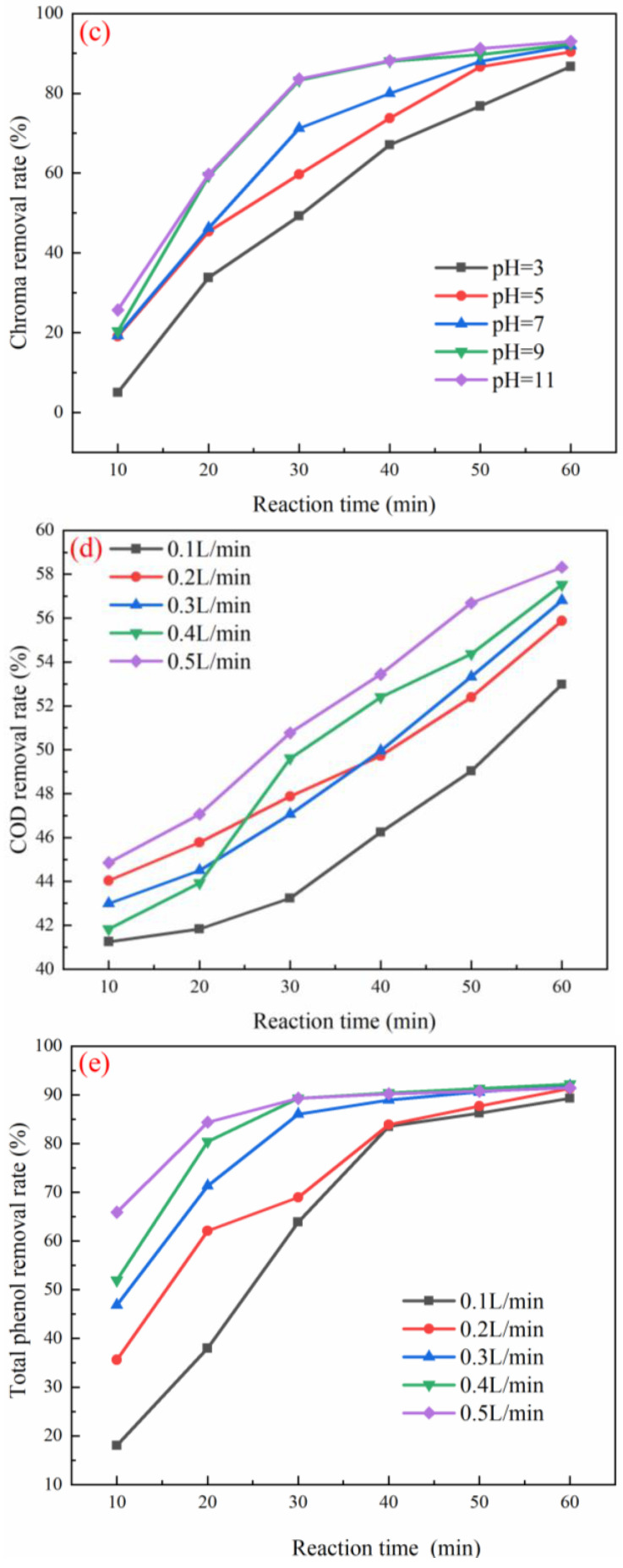

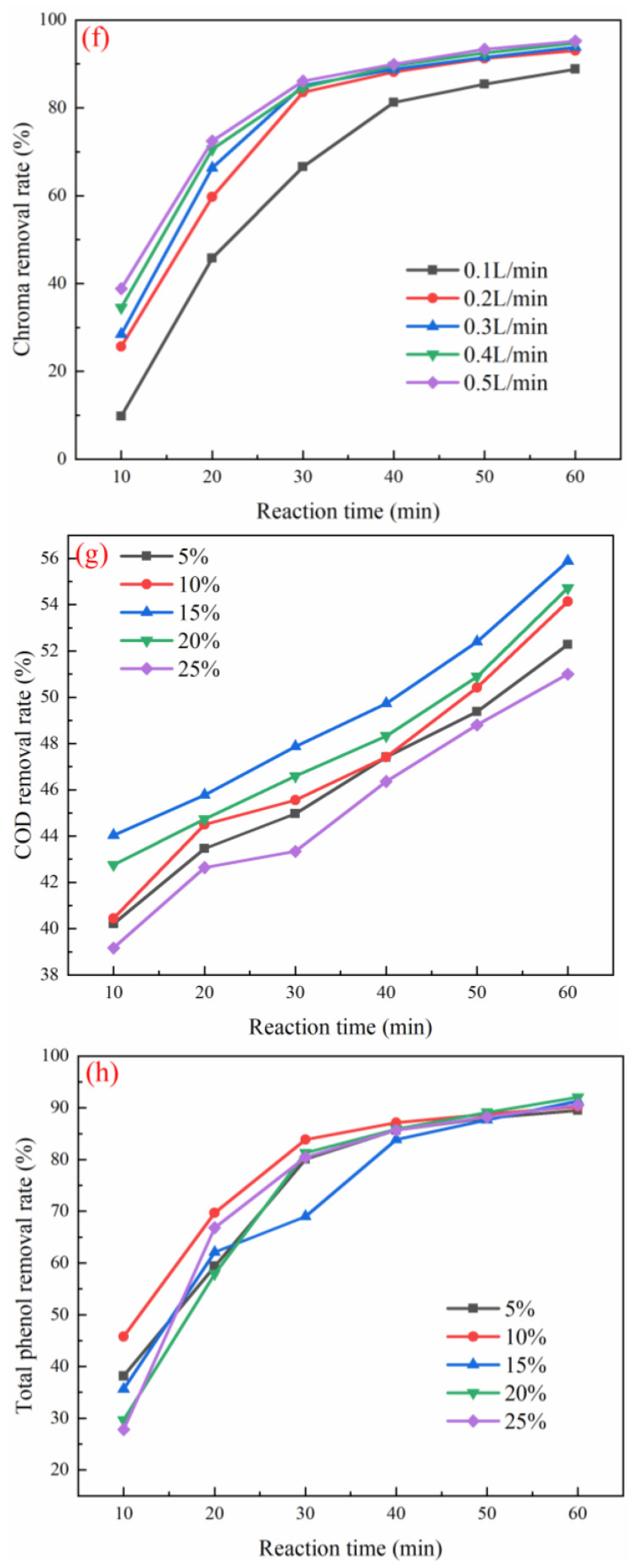

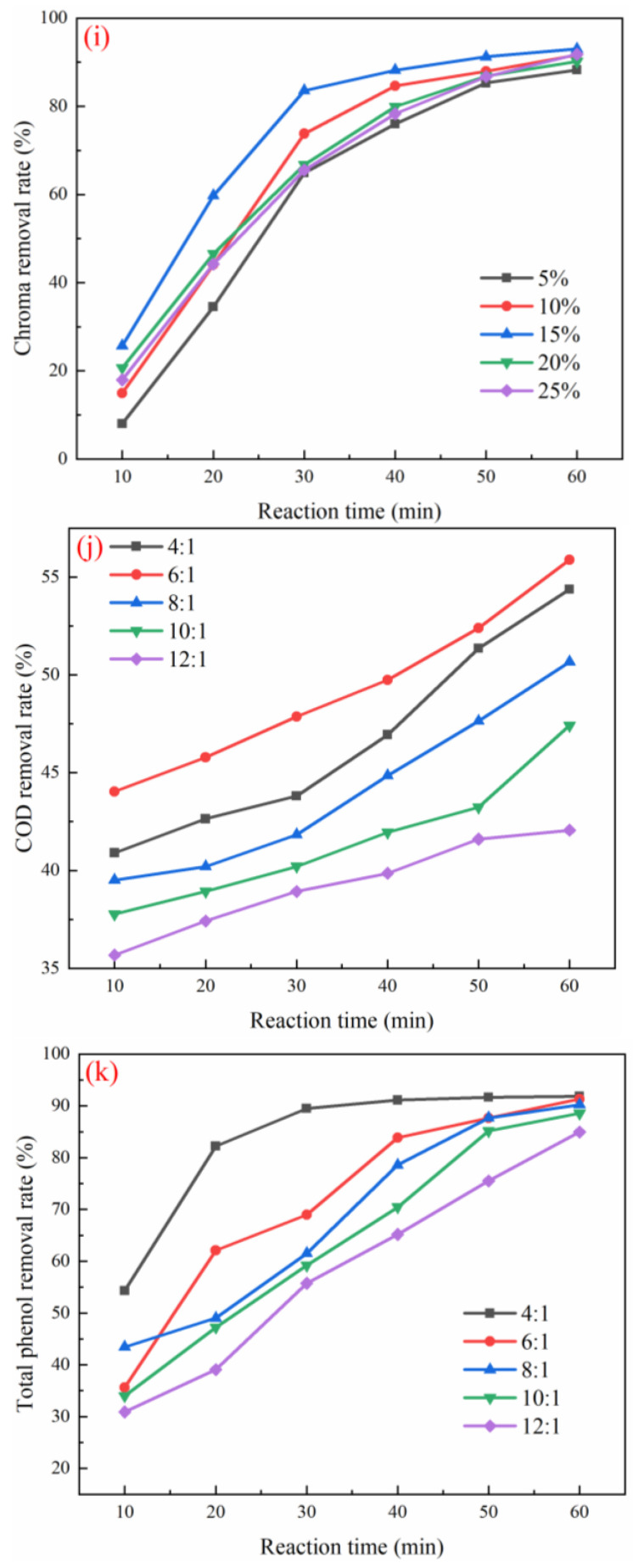

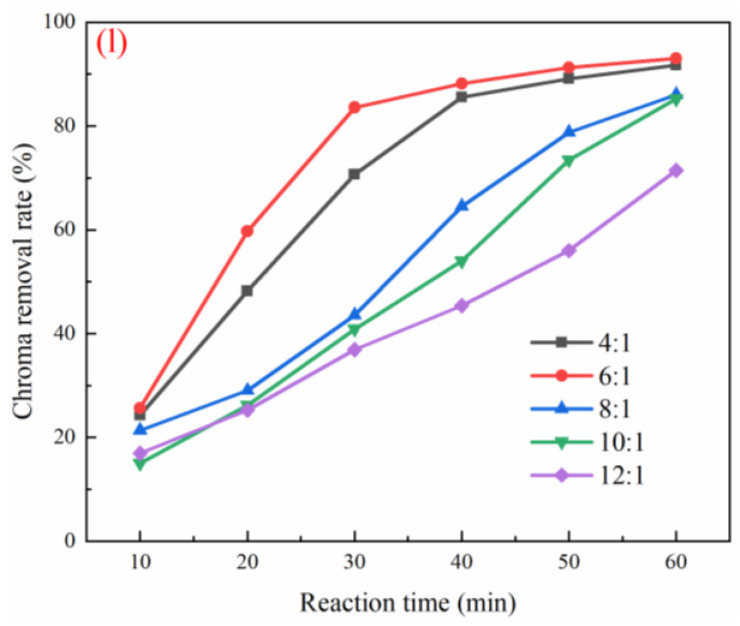
Effect of pH (**a**–**c**), gas flow rate (**d**–**f**), catalyst filling ratio (**g**–**i**), height to diameter (**j**–**l**) on removal performance of COD, total phenol, and chroma.

The effect of pH on pollutants removal was performed with an ozone flow rate of 0.5 L/min, catalyst filling rate of 15% and H/D of 6. As shown in Figure 3a–c, when the wastewater pH = 11, the optimal removal rates of COD, total phenol, and color were 56.6%, 93.9%, and 92.5%, respectively. When the pH was 3–9, the COD removal rate increased from 46.2% to 54.9%. When the pH was 9–11, the COD removal rate increased from 54.9% to 56.6%. At pH = 3–9, the removal rates of total phenol and color increased from 89.6% and 86.7% to 91.8% and 92.3%, respectively. At pH = 9–11, the removal rates of total phenols and chroma increased from 91.8% and 92.3% to 92.5% and 93.9%, respectively. Under alkaline conditions, a large amount of OH- in wastewater would react rapidly with ozone and convert to sufficient •OH, which was more conducive to the rapid degradation of pollutants. As shown in Table 1, the raw water pH of coal chemical wastewater was 9.28, and the alkaline environment was conducive to catalytic ozonation reaction. There is no need to adjust the pH, which reduces the treatment cost and avoids the introduction of new ions. The raw water pH = 9.28 was selected as the optimal working condition [20].

The effect of the ozone flow rate on pollutants removal was conducted with pH = 9, catalyst filling rate of 15%, and H/D of 6. As shown in Figure 3d–f, when the gas flow was increased from 0.1 to 0.5 L/min, the COD removal rate increased from 52.9% to 58.3% and chroma removal rate increased from 88.2% to 93.0%. The best removal rate of total phenol was 92.2% at 0.4 L/min, but the removal rate of total phenol decreased to 91.4% when the gas flow was 0.5 L/min. In the catalytic oxidation reaction, with the increase of gas flow rate, the driving force of mass transfer between the gas–liquid systems was greater, mixing of ozone and wastewater was sufficient, and degradation efficiency of organic matter in wastewater also increased. The pressure will be provided, making the catalyst active site more fully contacted with ozone, thereby improving the degradation efficiency of pollutants in the ozone catalytic oxidation system [21]. However, excessive gas flow will cause the catalyst to wear during the reaction process and shorten the service life of the catalyst. Therefore, the gas flow rate of 0.2 L/min was selected as the optimal working condition.

The effect of catalyst filling rate on pollutants removal was carried out with pH = 9, an ozone flow rate of 0.5, and H/D of 6. As shown in Figure 3g–i, with the increase of the catalyst filling rate, the removal rates of COD, total phenol, and chromaticity all showed a trend of first increasing and then decreasing. When the catalyst filling rate reached 20%, the removal rates of COD and chromaticity reached the highest, 55.8% and 93.0%, respectively [22]. The best removal rate of total phenol was 91.1% when the catalyst filling rate was 25%. However, the excess catalyst would cover each other and hinder the contact between the active site, ozone, and wastewater [23]. This condition further reduced the degradation efficiency of the catalytic ozone oxidation system. In addition, if a large amount of catalyst is added, a large amount of •OH will aggregate to produce a quenching effect, resulting in a decrease in the utilization efficiency of •OH [24]. The catalyst filling rate of 20% was selected as the optimal working condition.

The impact of H/D ratio on pollutants removal was performed with pH = 9, an ozone flow rate of 0.5, and a catalyst filling rate of 15%. As shown in Figure 3j–l, when the height–diameter ratio of the reactor was increased from 4:1 to 6:1, the COD removal rate increased from 54.3% to the highest 55.8%, and the color removal rate increased from 91.7% to the highest 93.0%. When the height–diameter ratio was 6:1–12:1, the removal rate of COD decreased from 55.8% to 42.0%, and the removal rate of chroma decreased from 93.0% to 71.4%. When the height–diameter ratio was 4:1–12:1, the total phenol removal rate decreased from 91.8% to 84.9%. When the height and diameter were small, the residence time of the gas in the reaction tower was very short, and the direct oxidation reaction of ozone was the main reaction in the system at this time. With the increase of the aspect ratio, the residence time of ozone in the water became longer, and the ozone had enough time to contact the catalyst and organic matter. At this time, the indirect degradation reaction of •OH in the system dominated [10]. However, if the height–diameter ratio was too high, it meant a larger amount of wastewater treatment and catalyst dosage, resulting in a large increase in the content of organic matter and treatment costs. With these overall considerations, the height–diameter ratio of the reaction tower of 6:1 was selected as the best working condition for the experiment.

### 3.4. Stability Analysis of Mn/Zn@PG Ozone Catalyst

As shown in Figure S9, the wear rate of the catalyst increased from 4.5% to 9.1% when the rotation speed was between 40 and 120 rpm. When the rotation speed was between 120 and 140 rpm, and the wear rate of the catalyst increased from 9.1% to 12.3%. At a higher rotation speed, the surface of the catalyst produced severe friction and wear during the reaction. However, in the actual wastewater treatment, the wear of the catalyst caused by the impact of water flow was much smaller than the oscillatory wear caused by the experiment, so the Mn/Zn@PG catalyst met the application requirements for practical engineering. As shown in Figure S10, under the optimal working conditions, the COD removal rate of the first use of the Mn/Zn@PG catalyst to treat coal chemical wastewater reached 55.8%. Thereafter, the COD removal rates dropped to 50.6%, 47.4%, 44.4%, 42.4%, and 41.3%. This result indicates that the Mn/Zn@PG catalyst undergoes passivation after repeated use, which may be caused by the loss of active components on the surface of the catalyst, caused by mutual friction during the use of the catalyst and blockage of the catalyst pore size by macromolecular organic matter [25]. As shown in Table 4, after 60 min treatment, the contents of the Mn and Zn elements in the wastewater reached 15.52 and 14.27 mg/L, respectively, and the leached Zn and Mn accounted for 6.7% and 11.9% of the total metal content, respectively. After 60 min of treatment, the chromaticity removal rate of the wastewater was high, but the treated wastewater still showed a light brownish yellow color. The reason was that the wastewater contained incompletely treated organic matter, and metal ions were dissolved in the catalyst during the treatment process. The high dispersion of metal oxide crystals in the Mn/Zn@PG catalyst and the strong interaction between Zn and Mn bimetals can inhibit the leaching of metal oxides. However, during the reaction process, with the increase of phenolic macromolecules in wastewater, the leaching of metal oxides can be suppressed. The decomposition produces intermediates, such as organic acids, which reduce the pH of the wastewater, thereby leading to the dissolution of a small amount of metal elements in the catalyst [26].

### 3.5. Effect of Dosage of Radical Quencher on Different Reaction Systems

**Figure 4:**
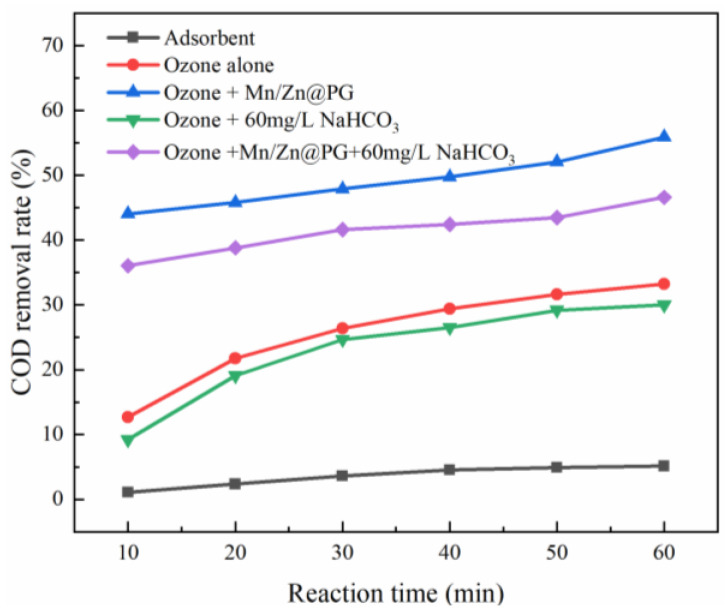
COD removal rates under different reaction systems.

Figure 4 shows that the COD removal rate contributed by the carrier adsorption is only 5.1%. In the single O_3_ system, the COD removal rate was 33.2%; in the O_3_+Mn/Zn@PG catalyst system, the COD removal rate reached 55.8%, an increase of 22.6%, compared with that in the single O_3_ system. A large amount of O_3_ occurs in both systems, but the difference is that the addition of Mn/Zn@PG catalyst promoted the decomposition of O_3_, so that the O_3_+catalyst system contained a large amount of •OH [27].

As shown in Figure 4, after 60 mg/L of NaHCO_3_ was added to the O_3_ system alone, the COD removal rate decreased to 29.9%. At this time, the direct oxidation reaction of O_3_ was the main reaction in the reaction system. After 60 mg/L of NaHCO_3_ was added to the O_3_+catalyst system, the removal rate of COD decreased to 46.5%, indicating the existence of a large amount of •OH in the reaction system at this time [27]. After a radical quencher was added, the quencher not only captured •OH in the reaction system but also occupied the active sites of the catalyst support, resulting in a decrease in the COD removal rate in the reaction system [28].

### 3.6. Analysis of UV Absorption Peaks of Wastewater before and after Reaction

**Figure 5:**
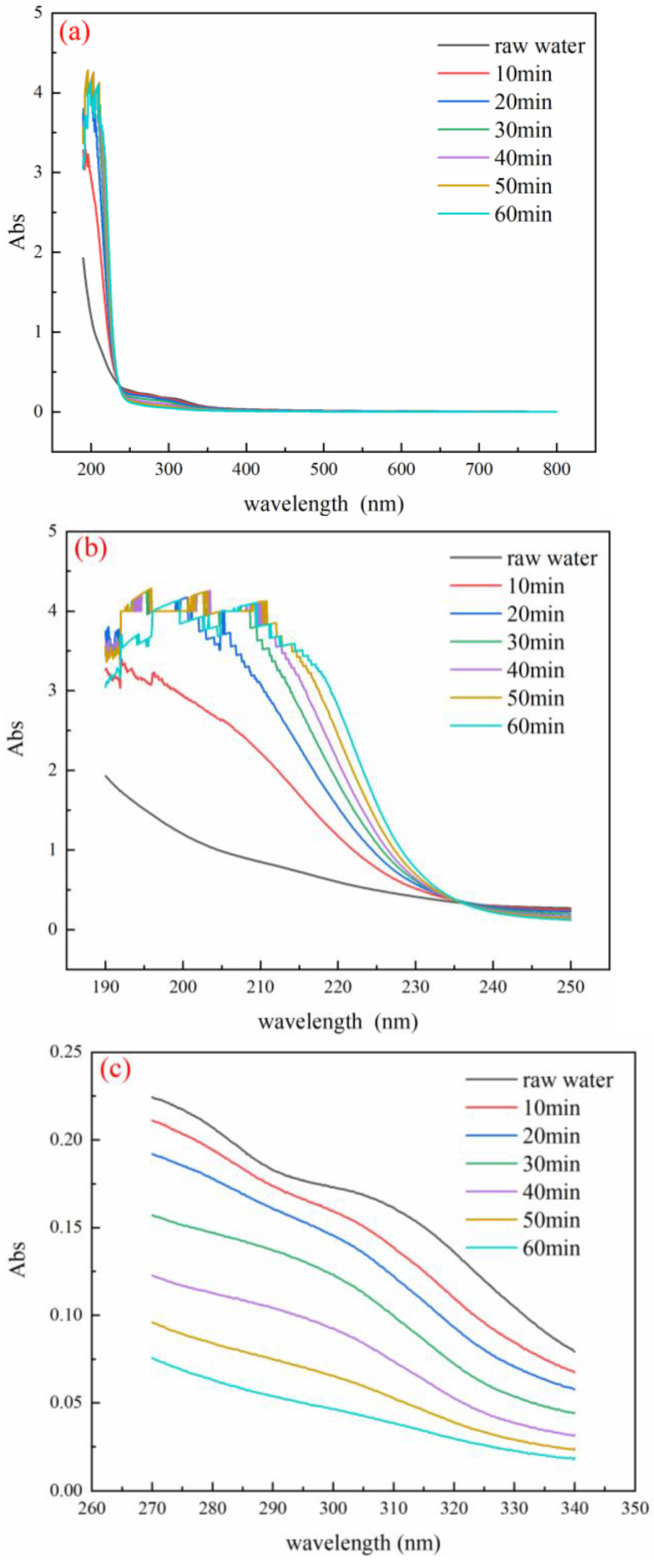
UV–visible spectrum of coal chemical wastewater: (**a**) 190–800 nm; (**b**) 190–250 nm; (**c**) 270–340 nm.

As shown in Figure 5b, an obvious absorption peak occurs in the 200–230 nm band, where the peak is usually related to organic acids and aromatic protein compounds [29]. As shown in Figure 5c, an obvious absorption peak occurred at 300 nm. The absorption peak in this section is related to organic compounds such as π-π double bonds, indicating that the wastewater contains benzene series organic compounds, polycyclic aromatic hydrocarbons, and other substances [30]. With the increase of the treatment time, the intensity of the absorption peak in this section decreased. This result indicates that the benzene-based organic compounds and polycyclic aromatic hydrocarbons in the wastewater were effectively removed when the reaction was carried out for 60 min and decomposed into small molecular organic compounds or directly mineralized into CO_2_ and H_2_O. With the increase of treatment time, the number of organic acids and aromatic protein compounds in the wastewater increased instead. The reason is that a large number of macromolecular benzene-based organic compounds and phenol contained in the raw water decomposed into a large number of organic acids and other small molecules. For organic matter, when the reaction time reaches 60 min, the amount of organic acids and aromatic protein compounds began to decrease.

### 3.7. 3D Fluorescence Spectroscopic Analysis of Wastewater before and after Reaction

**Figure 6:**
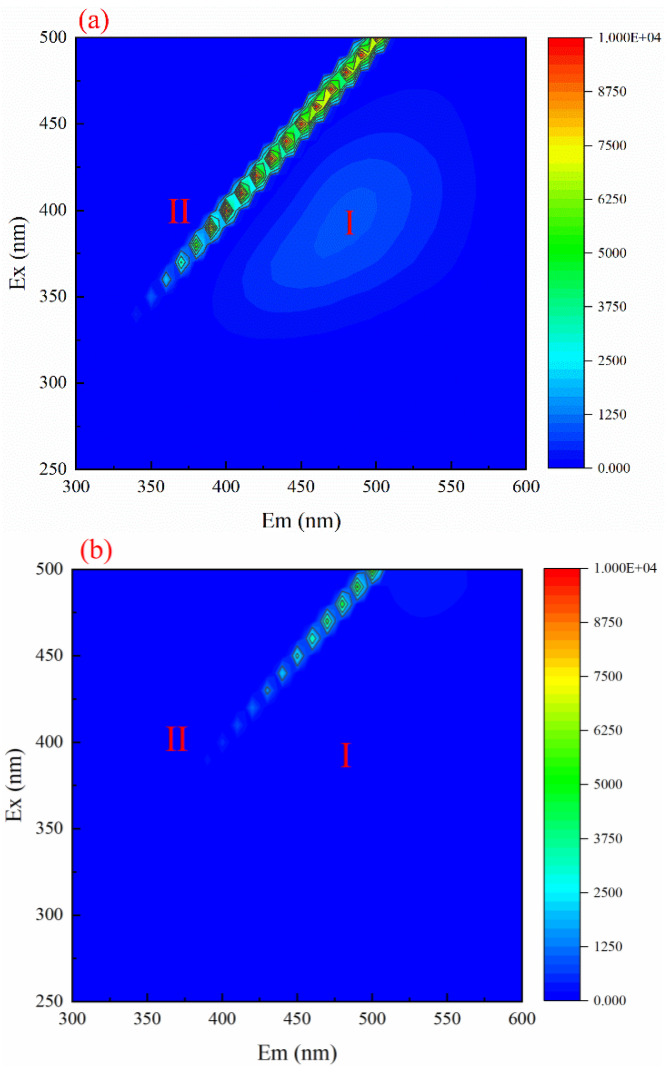
The 3D fluorescence spectra of coal chemical wastewater under different conditions: (**a**) before treatment; (**b**) after processing.

Two main regions, peak I (Ex/Em: 350–400/450–550) and peak II (Ex/Em: 350–500/350–525), are shown in Figure 6a. The peaks I and II regions were the highest. The fluorescence intensities were 1500 and 10,000, respectively, which indicated that soluble microbial products (SMP) and humic acids were the main organic pollutants in coal chemical wastewater [31]. Figure 6b does not observe the existence of peak I and only has a regional peak II (Ex/Em: 280–550/270–490), which indicates that after ozone catalytic oxidation treatment, part of the SMP, humic acids, and polycyclic aromatic hydrocarbon humic acids decomposed into a small amount of aromatic compounds, organic acids, and other small molecular organic compounds [32].

### 3.8. Effect of Various Reaction Systems on Ozone Utilization

**Figure 7:**
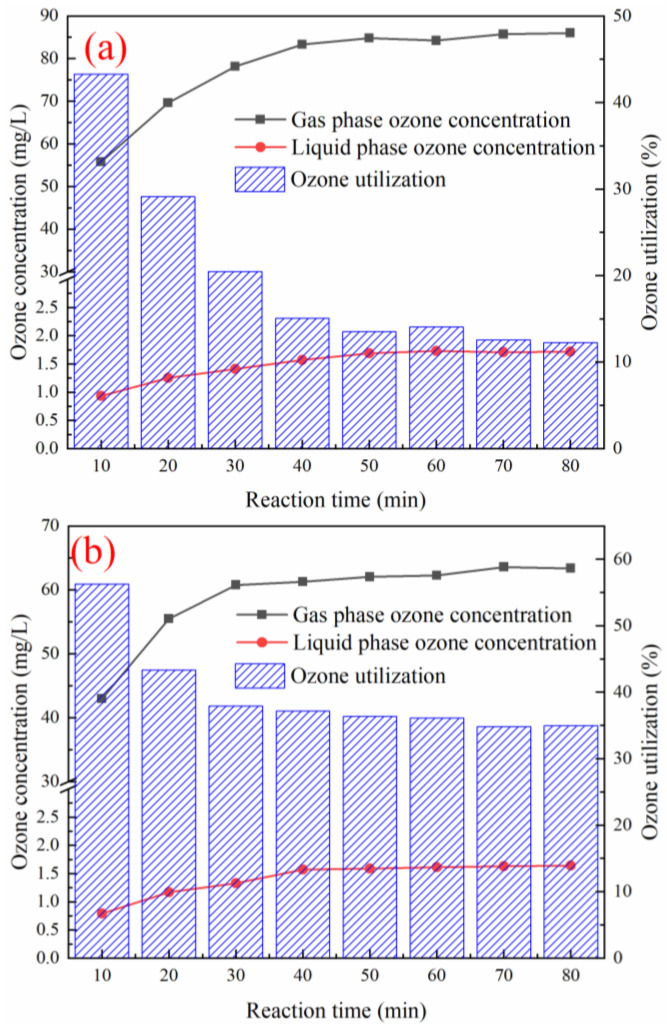
Ozone utilization under different reaction conditions: (**a**) ozone alone; (**b**) O_3_+Mn/Zn@PG.

As shown in Figure 7, in the single O_3_ system, the highest ozone utilization rate was 43.3%, when the reaction was carried out for 10 min, and the ozone utilization rate was stable at 13.5%, when the reaction was conducted for about 50 min. In the O_3_+Mn/Zn@PG catalyst system, the highest ozone utilization rate was 56.2%, when the reaction proceeded for approximately 10 min, and the ozone utilization rate in the reaction system was stable at 37.1%, when the reaction proceeded for approximately 40 min. This is because in the initial stage, ozone will cooperate with Mn/Zn@PG catalyst, or ozone itself will decompose to generate a large number of •OH to react with pollutants [33]. When the reaction was carried out for 60 min, the ozone utilization rate of the O_3_ system alone and O_3_+Mn/Zn@PG catalyst system reached 14.0% and 36.1%, respectively. A comparison of the two groups of experiments shows that, after the Mn/Zn@PG catalyst was added, the utilization rate of ozone in the reaction system increased by 22.0%.

### 3.9. Analysis of Comprehensive Evaluation Model of Ozone Catalytic Oxidation Experimental Hierarchy–Principal Component

**Figure 8:**
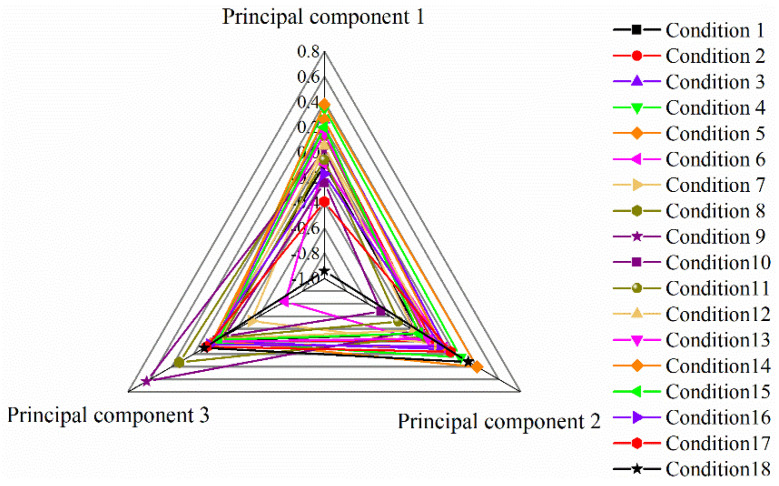
Analysis diagram of comprehensive evaluation.

The comprehensive evaluation analysis results are shown in TEXT S2 and Figure 8. The results show that the comprehensive evaluation scores of working conditions 5, 4, 3, 14, 2, and 15 were much higher than other groups. The comprehensive evaluation score of working condition 5 with the highest score is 0.27; the higher the score, the better the comprehensive effect of the working condition. The experimental conditions of this group are as follows: reaction time 60 min, wastewater pH = 9.28, gas flow rate 0.5 L/min, and catalyst filling rate 15%. At this time, the removal rates of COD, total phenols, and chromaticity of the wastewater reached 58.3%, 91.4%, and 95.2%, respectively. The environmental benefits were high, and the cost consumption and energy consumption remained relatively low. Therefore, the fifth group of working conditions was the best operating conditions for this experiment.

## 4. Conclusions

In this study, palygorskite was used as the carrier, and two metal elements, Zn and Mn, were selected to prepare Mn/Zn@PG catalyst via the doping-calcination method. The optimal preparation conditions were as follows: for Zn:Mn = 1:2, the calcination temperature was 400 °C and time was 4 h. The characterization results showed that the optimal calcination temperature of Mn/Zn@PG catalyst was 400 °C, and the Mn and Zn elements were successfully loaded in the catalyst. After 20 uses, the content of the active components in the catalyst did not decrease significantly. The pores in the catalyst were mainly mesopores, and the Zn and Mn elements contained in them existed in the form of ZnO and MnO_2_ metal oxides. The optimal working conditions for Mn/Zn@PG catalyst to degrade coal chemical wastewater were the following: wastewater pH = 9.28, gas flow rate 0.2 L/min, catalyst filling rate 20%, and reaction tower height–diameter ratio 6:1. After the catalyst was used 20 times, the COD removal rate in the reaction system remained at 41.3%. The results of UV–vis spectroscopy and 3D fluorescence spectroscopy indicated that a large number of macromolecular organic compounds, such as phenols and polycyclic aromatic hydrocarbons, were decomposed in the raw water. When NaHCO_3_ was added to the separate ozone and catalytic ozone oxidation systems, the COD removal rate in the separate O_3_ system decreased to 29.9%, and the COD removal rate in the O_3_+catalyst system decreased to 46.5%. Under the same conditions, the ozone utilization rate of the catalytic ozone oxidation system and separate ozone system were 36.1% and 14.0%, respectively. The optimal working conditions of the experiment obtained by constructing the hierarchical–principal component comprehensive analysis method were the following: reaction time 60 min, wastewater pH = 9.28, gas flow rate 0.5 L/min, and catalyst filling rate 15%.

## Figures and Tables

**Table 1:** Water quality indicators and measurement methods.

Index	Measurement Methods	Unit	Content
COD	Dichromate method	mg/L	8613
Total phosphorus (TP)	Ammonium molybdate spectrophotometry	mg/L	895
Total nitrogen (TN)	Alkaline potassium persulfate digestion UV spectrophotometry	mg/L	400
NH_3_-N	Nessler’s reagent colorimetry	mg/L	234
pH	Glass electrode method	/	9.28
Conductivity	Glass electrode method	μs/cm	1709
Turbidity	Turbidimeter method	NTU	10.4
Chroma	Dilution factor method	PCU	6425
Total phenols	4-aminoantipyrine spectrophotometry	mg/L	790

**Table 2:** XRF characterization results.

Material	SiO_2_	MgO	Al_2_O_3_	Na_2_O	Fe_2_O_3_	CaO	ZnO	MnO_2_
Palygorskite blank sample	58.9072	13.5673	10.2627	6.8231	5.6022	1.8756	0.0149	0.1201
Mn/Zn@PG catalyst	51.4735	12.2776	9.0871	2.0537	5.2602	1.1365	5.4701	10.6499
Mn/Zn@PG catalyst reused 20 times	48.3199	11.7355	8.4605	7.0514	5.1028	1.2170	5.3694	10.3719

**Table 3:** EDS characterization of Mn/Zn@PG catalyst.

Sample	Palygorskite		Mn/Zn@PG		Mn/Zn@PG after Utilization 20 Times	
Element	Weight percentage	Atomic percentage	Weight percentage	Atomic percentage	Weight percentage	Atomic percentage
OK	55.89	68.95	48.97	64.93	57.62	70.95
MgK	9.37	7.61	8.00	6.98	9.72	7.88
AlK	5.01	3.67	5.52	4.34	3.74	2.73
SiK	26.57	18.68	25.69	19.40	23.81	16.70
FeK	2.68	0.95	2.94	1.12	1.73	0.61
MnK	0.11	0.04	5.65	2.18	1.86	0.67
ZnK	0.35	0.11	3.24	1.05	1.51	0.46

**Table 4:** The content of metallic elements in water sample.

Elements	Mn (mg/L)	Zn (mg/L)
Raw water	0	0
Water sample after oxidation treatment	15.52	14.27

## Data Availability

The data presented in this study are available on request from the corresponding author.

## Supplementary Materials

The following supporting information can be downloaded at: https://www.mdpi.com/article/10.3390/ijerph191710812/s1. Figure S1: Comprehensive evaluation index system of Mn/Zn@PG catalyst. Figure S2: Effect of element doping ratio on COD and chromaticity removal performance: (a) COD. (b) chroma. Figure S3: Effect of calcination temperature on COD and chromaticity removal performance: (a) COD; (b) chroma. Figure S4: Effect of calcination time on COD and chromaticity removal performance: (a) COD; (b) chroma. Figure S5: XRD characterization of the catalyst. Figure S6: Adsorption and desorption isotherms: (a) blank; (b) Mn/Zn@PG catalyst. (c) Mn/Zn@PG catalyst used 20 times. (d) Pore size distribution. Figure S7: EDS characterization results: (a) blank; (b) Mn/Zn@PG; (c) Mn/Zn@PG after utilization 20 times. Figure S8: Peak fitting diagram of catalyst: (a) Zn2p; (b) Mn2p. Figure S9: Wear rate of Mn/Zn@PG catalyst. Figure S10: Effect of multiple use on catalyst stability. Table S1: The value standard of RI. Table S2: Judgment matrix of evaluation layer to target layer. Table S3: The judgment matrix of environmental benefit in index level. Table S4: The judgment matrix of cost consumption in index level. Table S5: The judgment matrix of energy consumption in index level. Table S6: The total ranking weight of each index layer. Table S7: Summary of experimental data. Table S8: Experimental data standardization. Table S9: Correlation coefficient matrix. Table S10: Index characteristic values and variance explanation rate. Table S11: Principal component load coefficients. Table S12: Comprehensive score coefficients of principal components. Table S13: BET characterization results of catalysts. Table S14: Ranking of comprehensive scores. Text S1: Establishment of evaluation model. Text S2: Evaluation model results.
