# Supplementary material for: Preparation of Mn/Zn@PG Catalyst for Catalytic Oxidation Treatment of Coal Chemical Wastewater
Source: Int J Environ Res Public Health. 2022 Aug 30;19(17):10812. doi: 10.3390/ijerph191710812 (PMC9518122; doi:10.3390/ijerph191710812)
Supplement: Supplementary file 1 [file ijerph-19-10812-s001.zip › ijerph-1852240-supplementary.pdf]

TEXT S1 Establishment of evaluation model

In order to realize the comprehensive evaluation of Mn/Zn@PG catalytic ozonation of coal chemical wastewater, it is necessary to select comprehensive evaluation indicators and build relevant evaluation index system. Analytic hierarchy process (AHP) is a general comprehensive evaluation theory, which was developed by Saaty in the 1980s and used in the decision-making of problems involving multiple goals, principles or levels. AHP simplifies the complex system into a one-to-one comparison between various indicators, and then obtains the weight of indicators. Principal component analysis (PCA) is to use the method of dimension reduction to gather the indicators with high correlation into a principal component, and a few principal components represent all the indicators that are originally complex and diverse, making the problem easier to understand and analyze. In this study, AHP-PCA comprehensive evaluation model is established to analyze the data obtained in the test. Through the dual combination of these two models, the accuracy of the evaluation results can be effectively improved. It can more effectively solve the problems such as the compatibility of the method set, random error, systematic deviation of the final evaluation results, so as to comprehensively improve the accuracy, reliability and effectiveness of the research.

1. Calculation of index weight and consistency test

According to the selected specific evaluation indicators, a comprehensive evaluation index system for catalytic ozonation of coal chemical wastewater is established, and the structure is shown in Figure S1

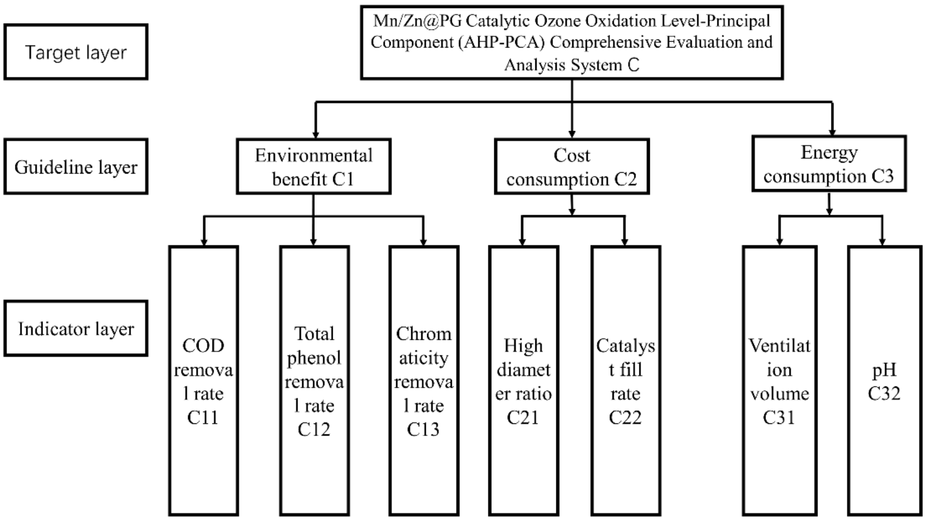

Figure S1. Comprehensive evaluation index system of Mn/Zn@PG catalyst.

According to the 1-9 scale method, the importance of each factor is determined to construct a judgment matrix, through which the weight of each index between each single level is determined and the consistency is judged.

The calculation of single level index sorting is shown in function 1 to 3:

$$Ni = \prod_{j=1}^{n} aij \quad (i = 1,2 \ldots, n) \tag{S1}$$

$$Mi = (Ni)^{\frac{1}{n}} \tag{S2}$$

$$Mi^{*} = \frac{Mi}{\sum_{j=1}^{n} Mj} \tag{S3}$$

Where：Ni：Product of row elements in judgement matrix;

Mi*：Single level sorting weight corresponding to an indicator.

Consistency test is shown in function 4 to 5：

$$CI = \frac{\lambda_{max} - n}{n-1} \tag{S4}$$

$$CR = \frac{CI}{RI} \times 100\% \tag{S5}$$

Where, λ_{max}: Maximum eigenvector of matrix layer;

CR: Consistency test results;

RI: Consistency index；

n: Judgement matrix order.

CR=0 indicates the complete consistency of the judgement matrix. The greater the CR, the worse the consistency. The value of RI is shown in Table S1.

Table S1. The value standard of RI.

n	1	2	3	4	5	6	7	8	9
RI	0	0	0.58	0.90	1.12	1.14	1.32	1.41	1.45

The judgment matrix of criterion layer and index layer and the weight of each single-layer factor can be obtained using the method above, and the consistency judgment result can be obtained. The index ranking results are shown in Tables S2-S5.

Table S2. Judgment matrix of evaluation layer to target layer.

**N**	**C1**	**C2**	**C3**	**Weight M1**
Environmental benefit C1	1	2	2	0.50
Cost consumption C2	1/2	1	1	0.25
Energy consumption C3	1/2	1	1	0.25
Consistency test	λ_{max}=3, CI=0, CR=0, Completely consistent			

Table S3. The judgment matrix of environmental benefit in index level.

C1	C11	C12	C13	Weight M2
COD removal rate C11	1	1	1	0.33
Total phenol removal rate C12	1	1	1	0.33
Chromaticity removal rate C13	1	1	1	0.33
Consistency test	λ_{max}=3, CI=0, CR=0, Completely consistent			

Table S4. The judgment matrix of cost consumption in index level.

C2	C21	C22	Weight M2
H/D C21	1	1	0.50
Catalyst fill rate C22	1	1	0.50
Consistency test	λ_{max} =2, CI=0, CR=0, Completely consistent		

Table S5. The judgment matrix of energy consumption in index level.

C3	C31	C32	Weight M2
Ventilation volume C31	1	2	0.66
pH C32	1/2	1	0.33
Consistency test	λ_{max} =2, CI=0, CR=0, Completely consistent		

The total ranking weight of each index at the index level is shown in Table S6.

Table S6. The total ranking weight of each index layer.

Criteria	Index	Single ranking weight M2	Total ranking weight M
Environmental benefit C1 (0.50)	COD removal rate	0.33	0.165
	Total phenol removal rate	0.33	0.165
	Chromaticity removal rate	0.33	0.165
Cost consumption C2 (0.25)	H/D	0.50	0.125
	Catalyst fill rate	0.50	0.125
Energy consumption C3 (0.25)	Ventilation volume	0.66	0.165
	pH	0.33	0.083

2. Standardization

The comprehensive evaluation and analysis of Mn/Zn@PG catalytic ozonation of coal chemical wastewater are carried out, and the data obtained from the optimization of working conditions in this paper are used. The specific data are summarized in Table S7.

The selected experimental data have different units and dimensions, which cannot be effectively evaluated and analyzed. In order to make full use of the experimental data information, the obtained data are standardized by using Z-score method. Suppose there are n evaluation samples, m evaluation indexes, and the standardized formula is as follows:

$$\overline{a_j} = \frac{\sum_{i=1}^{n} a_{ij}}{n} \quad \text{(S6)}$$

$$S_j = \sqrt{\frac{\sum_{i=1}^{n}\left(a_{ij}-\overline{a_j}\right)^2}{n-1}} \quad \text{(S7)}$$

$$a_{ij}^* = \frac{a_{ij}-\overline{a_j}}{S_j}\,(i = 1,2,\dots n, j = 1,2,\dots,m) \quad \text{(S8)}$$

Where, $\bar{a}_j$: Average value of evaluation index corresponding to the sample;

S_j: Variance of evaluation index corresponding to the sample;

a_{ij}^*: Standardized indexes.

The standardized data are shown in Table S7.

Table S7. Summary of experimental data.

Operating condition	COD removal rate (%)	Total phenol removal rate (%)	Chromaticity removal rate (%)	H/D	Catalyst fill rate (%)	Ventilation volume (L/min)	pH
1	52.98	89.30	88.82	6	15	0.1	9.28
2	55.88	91.11	93.00	6	15	0.2	9.28
3	56.81	91.84	93.81	6	15	0.3	9.28
4	57.51	92.20	94.77	6	15	0.4	9.28
5	58.32	91.48	95.20	6	15	0.5	9.28
6	52.28	89.48	88.26	6	5	0.2	9.28
7	54.14	90.21	91.62	6	10	0.2	9.28
8	54.72	92.02	90.19	6	20	0.2	9.28
9	51.00	90.57	91.80	6	25	0.2	9.28
10	46.24	89.66	86.70	6	15	0.2	3
11	50.89	90.21	90.38	6	15	0.2	5
12	52.98	90.75	91.95	6	15	0.2	7
13	54.95	91.84	92.38	6	15	0.2	9
14	56.69	92.56	93.94	6	15	0.2	11
15	54.37	91.84	91.75	4	15	0.2	9.28
16	50.66	90.21	86.04	8	15	0.2	9.28
17	47.41	88.57	85.23	10	15	0.2	9.28
18	42.06	84.95	71.44	12	15	0.2	9.28

3. Correlation matrix

In order to determine the correlation between indicators, it is necessary to determine the correlation coefficient matrix of indexes. The calculation formula is shown as follows:

$$R = \begin{bmatrix} r_{11} & r_{21} & \cdots & r_{1m} \\ r_{21} & \cdots & \cdots & r_{2m} \\ \vdots & \vdots & \vdots & \vdots \\ r_{n1} & r_{n2} & \cdots & r_{nm} \end{bmatrix} \quad \text{(S9)}$$

$$r_{ij} = \frac{\sum_{i=1}^{n} r_{ki} \cdot r_{kj}}{n-1} (i = 1,2,\ldots,n, j = 1,2,\ldots,m) \quad \text{(S10)}$$

Where, r_{ij}: Relationship coefficient between two standardized indexes.

The index relationship coefficient matrix is shown in Table S8.

Table S8. Experimental data standardization.

Operating condition	COD removal rate (%)	Total phenol removal rate (%)	Chromaticity removal rate (%)	H/D	Catalyst fill rate (%)	Ventilation volume (L/min)	pH
1	0.049	-0.666	-0.189	-0.310	0	-1.428	0.341
2	0.734	0.348	0.578	-0.310	0	-0.310	0.341

3	0.953	0.757	0.727	-0.310	0	0.807	0.341
4	1.118	0.958	0.902	-0.310	0	1.925	0.341
5	1.310	0.555	0.982	-0.310	0	3.042	0.341
6	-0.116	-0.565	-0.291	-0.310	-2.608	-0.310	0.341
7	0.323	-0.156	0.325	-0.310	-1.304	-0.310	0.341
8	0.460	0.858	0.063	-0.310	1.304	-0.310	0.341
9	-0.418	0.045	0.358	-0.310	2.608	-0.310	0.341
10	-1.542	-0.464	-0.578	-0.310	0	-0.310	-3.037
11	-0.444	-0.156	0.097	-0.310	0	-0.310	-1.961
12	0.049	0.146	0.385	-0.310	0	-0.310	-0.886
13	0.514	0.757	0.464	-0.310	0	-0.310	0.190
14	0.925	1.160	0.750	-0.310	0	-0.310	1.266
15	0.377	0.757	0.349	-1.428	0	-0.310	0.341
16	-0.498	-0.156	-0.699	0.807	0	-0.310	0.341
17	-1.266	-1.075	-0.847	1.925	0	-0.310	0.341
18	-2.528	-3.102	-3.377	3.042	0	-0.310	0.341

Table S9. Correlation coefficient matrix.

Index	COD removal rate (%)	Total phenol removal rate (%)	Chromaticity removal rate (%)	H/D	Catalyst fill rate (%)	Ventilation volume (L/min)
**COD removal rate (%)**	1	0.88	0.901	-0.723	-0.036	0.465
**Total phenol removal rate (%)**	0.88	1	0.927	-0.813	0.171	0.329
**Chromaticity removal rate (%)**	0.901	0.927	1	-0.846	0.079	0.372
**H/D**	-0.723	-0.813	-0.846	1	0	-0.102
**Catalyst fill rate (%)**	-0.036	0.171	0.079	0	1	0
**Ventilation volume (L/min)**	0.465	0.329	0.372	-0.102	0	1

4. Determine characteristic value and contribution rate

Calculate the characteristic value of the correlation coefficient matrix λ_j (j=1,2， …,p) and eigenvector e_j (j=1,2， …,p). Select the number of principal components P whose eigenvalue is greater than 1 (p≤m) and calculate the principal component contribution rate and cumulative contribution rate. The cumulative contribution rate of all selected principal components should be greater than 80%.

The calculation formula is as follows:

$$x_j=\frac{\lambda_j}{\sum_{k=1}^{m}\lambda_k}(j=1,2,\dots,m) \quad \text{(S11)}$$

$$Z_p=\frac{\overset{pi}{\sum_{i=1}^{i}}\lambda_k}{\sum_{k=1}^{m}\lambda_k}(j=1,2,\dots m) \quad \text{(S12)}$$

Where, x_j: Variance contribution rate of principal component y_i;

Z_p: Cumulative variance contribution rate of principal component y_1, y_2, …, y_p;

The eigenvalues, variance explanation rate and cumulative variance explanation rate of each component of the index layer are shown in Table S10:

Table S10. Index characteristic values and variance explanation rate.

Index	Eigenvalues			Principal components		
	**Eigenvalue**	**Variance explanation rate (%)**	**Cumulative variance explanation rate (%)**	**Eigenvalue**	**Variance explanation rate (%)**	**Cumulative variance explanation rate (%)**
COD removal rate	3.741	53.442	53.442	3.741	53.442	53.442
Total phenol removal rate	1.183	16.904	70.346	1.183	16.904	70.346
Chromaticity removal rate	1.018	14.542	84.888	1.018	14.542	84.888
H/D	0.848	12.114	97.002			
Catalyst fill rate	0.109	1.561	98.563			
Ventilation volume	0.065	0.932	99.495			
pH	0.035	0.505	100.00			

As shown in Table S10, there are three eigenvalues greater than 1, so three principal components (y_1, y_2 and y_3) are extracted. The variance explanation rates of principal components y_1, y_2 and y_3 were 53.442%, 16.904 and 14.542 respectively. The cumulative variance explanation rate of the principal components reached 84.888%, meeting the requirements

4. Determine load coefficient and comprehensive score coefficient of the principal component

The principal component load coefficient represents the relationship between the principal component and the index. The calculation formula of principal component load coefficient is as follows:

$$l_{ij} = \sqrt{\lambda_j} \cdot e_{ij} (i = 1,2,\cdots p, j = 1,2,\cdots,m) \quad \text{(S13)}$$

The formula for calculating the comprehensive score coefficient of the principal component is as follows:

$$d_{ij} = M_j \cdot \frac{l_{ij}}{\sqrt{\lambda_j}} \, (i = 1,2,\cdots p, j = 1,2,\cdots,m) \quad \text{(S14)}$$

Principal component load coefficient and comprehensive score coefficient are shown in Table S11 and S12:

Table S11. Principal component load coefficients.

Index	Load coefficients		
	**PC1**	**PC2**	**PC3**
COD removal rate	0.957	0.223	-0.065
Total phenol removal rate	0.957	-0.106	0.095
Chromaticity removal rate	0.971	-0.119	-0.015
H/D	-0.843	0.423	0.149
Catalyst fill rate	0.075	-0.194	0.975
Ventilation volume	0.449	0.417	-0.003
pH	0.221	0.847	0.177

Table S12. Comprehensive score coefficients of principal components.

Index	Comprehensive score coefficients		
	**PC1**	**PC2**	**PC3**
COD removal rate	0.084	0.062	-0.021
Total phenol removal rate	0.084	-0.030	0.031
Chromaticity removal rate	0.086	-0.033	-0.005
H/D	-0.056	0.089	0.037
Catalyst fill rate	0.005	-0.041	0.239
Ventilation volume	0.040	0.116	-0.001
pH	0.001	0.118	0.029

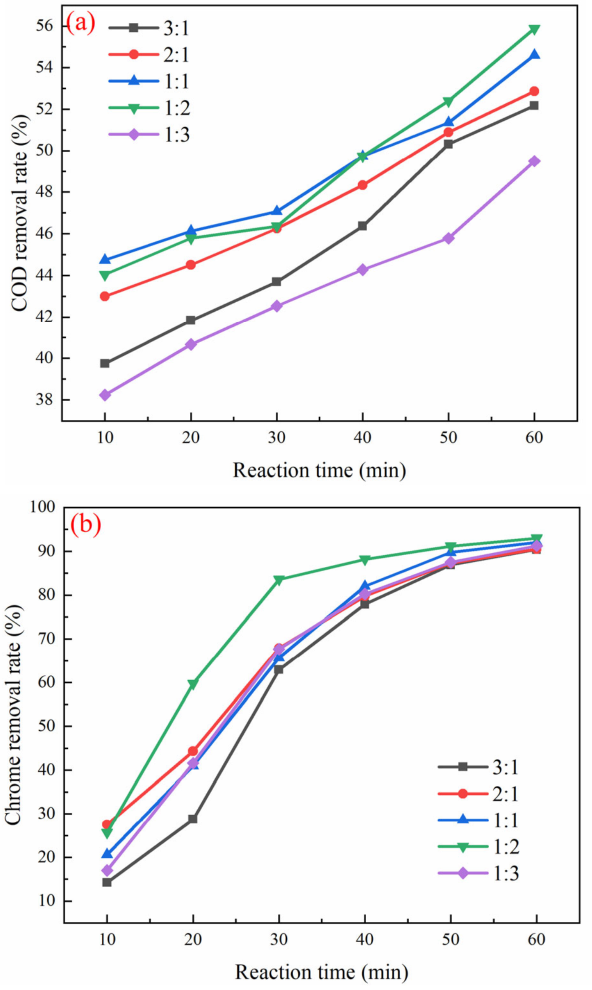

Figure S2. Effect of element doping ratio on COD and chromaticity removal performance: (a) COD; (b) chroma.

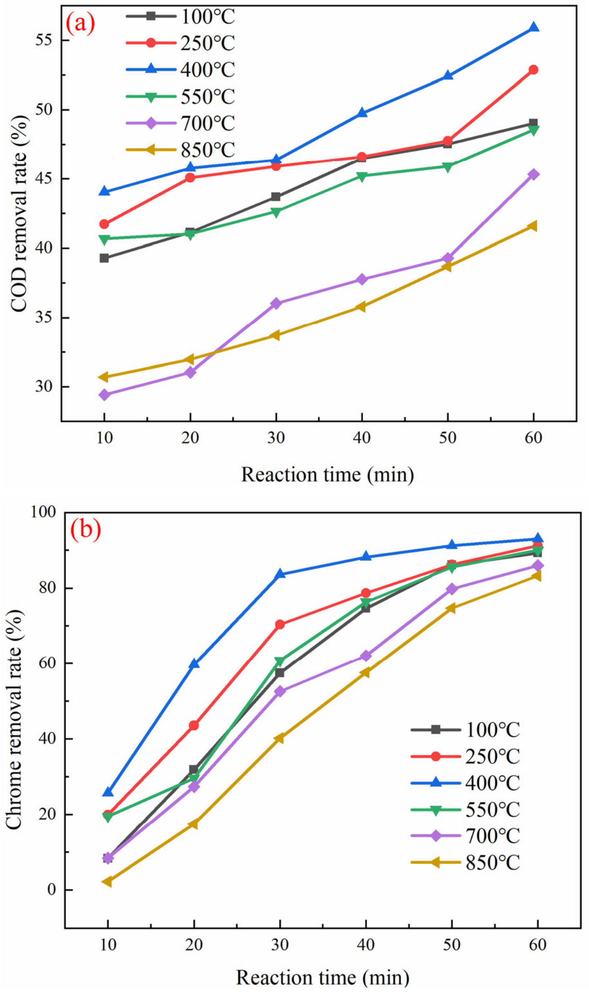

Figure S3. Effect of calcination temperature on COD and chromaticity removal performance: (a) COD; (b) chroma.

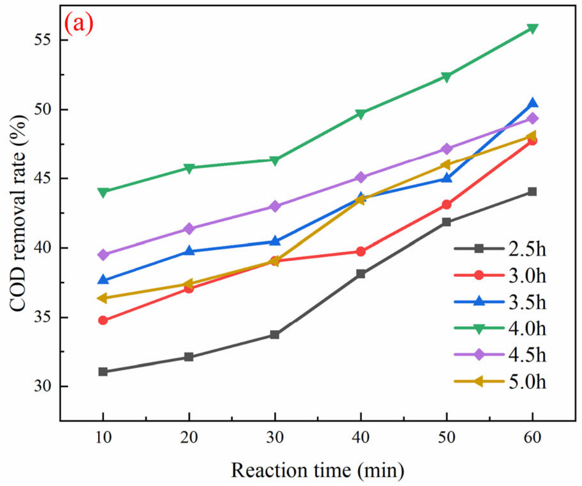

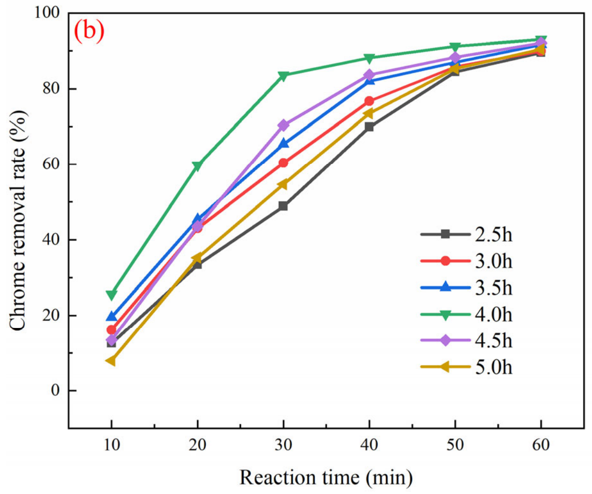

Figure S4. Effect of calcination time on COD and chromaticity removal performance: (a) COD; (b) chroma.

Characterization:

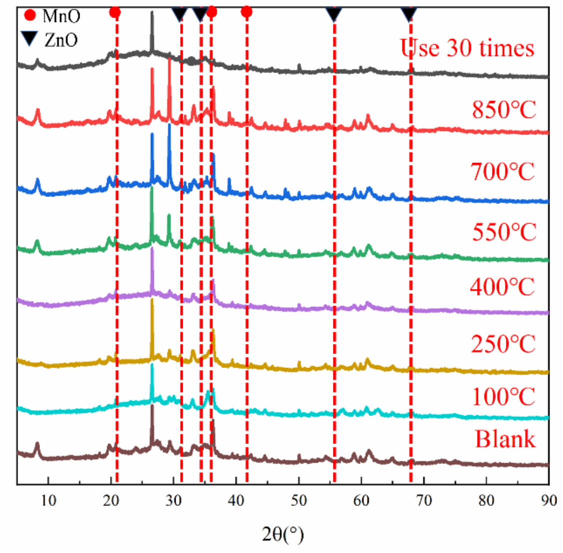

Figure S5. XRD characterization of the catalyst.

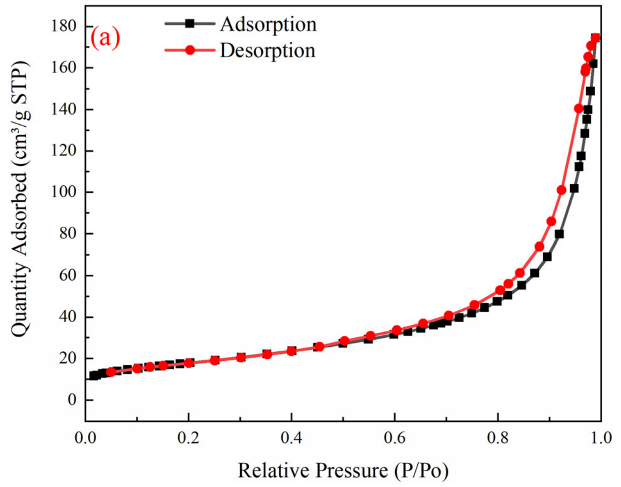

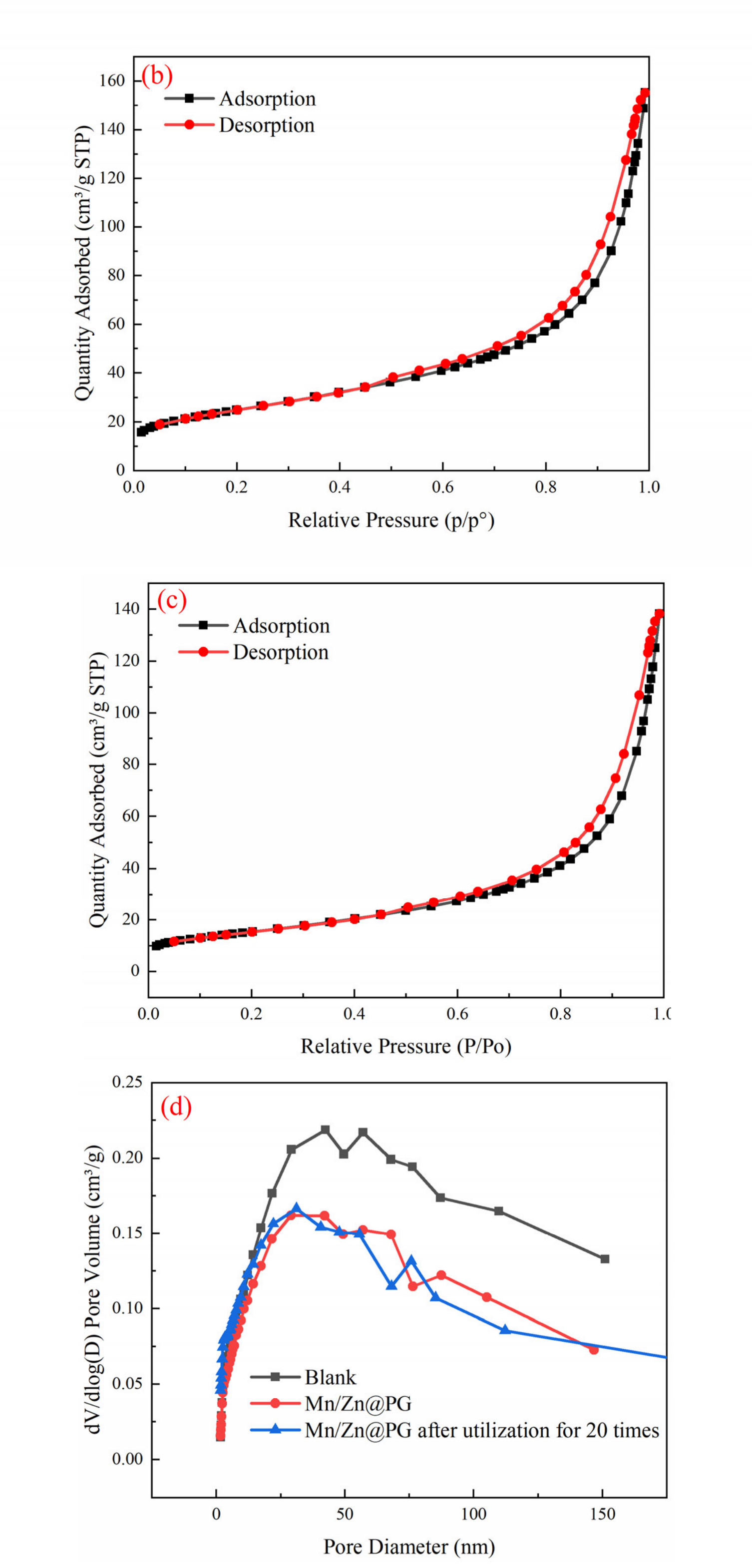

Figure S6. Adsorption and desorption isotherms :(a) Blank;(b) Mn/Zn@PG catalyst;(c) Mn/Zn@PG catalyst used 20 times;(d) Pore size distribution.

Table S13. BET characterization results of catalysts.

Sample	Specific surface area (m^2/g)	Average pore volume (cm^3/g)	Average pore size (nm)
Blank	63.3082	0.2685	15.2096
Mn/Zn@PG	54.5112	0.0146	13.8074
Mn/Zn@PG after utilization for 20 times	88.4639	0.2375	11.0474

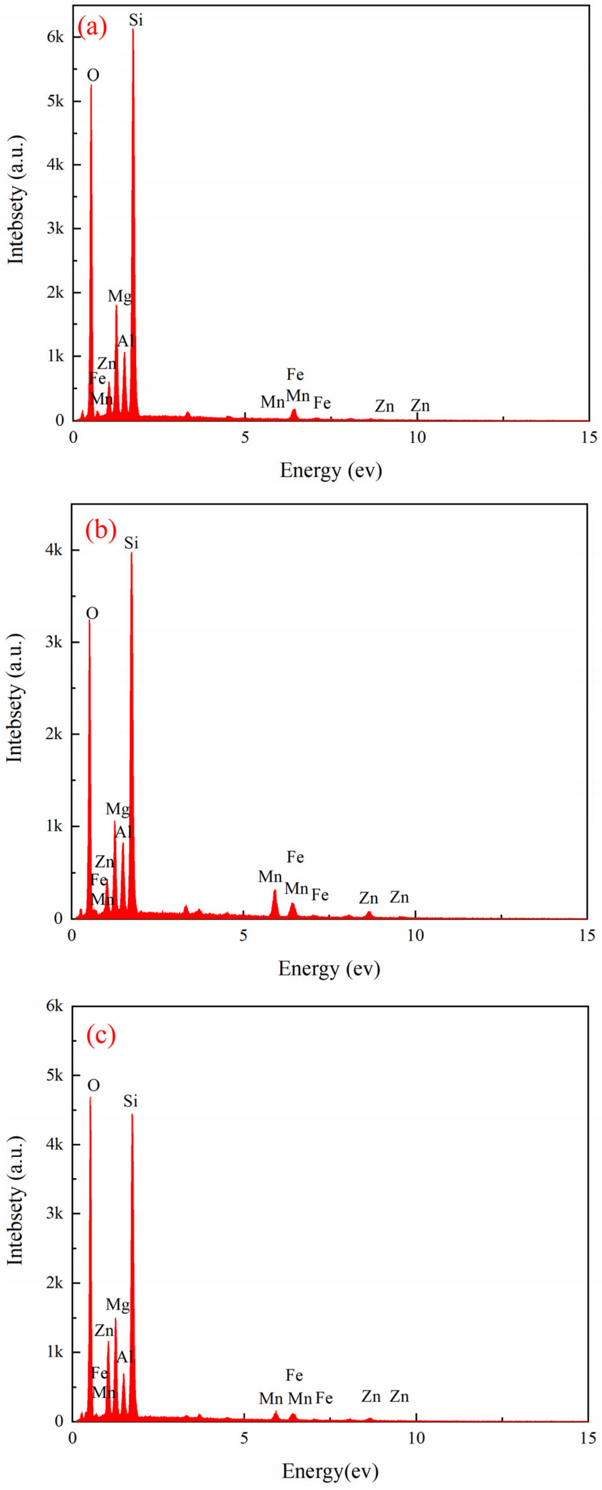

Figure S7. EDS characterization results :(a) Blankr;(b) Mn/Zn@PG;(c) Mn/Zn@PG after

utilization for 20 times.

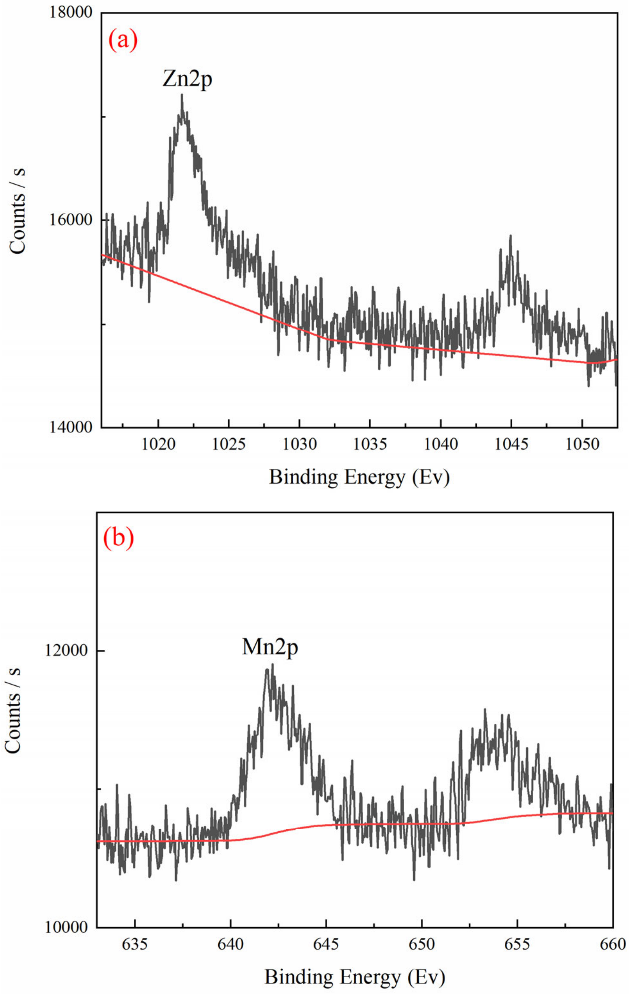

Figure S8. Peak fitting diagram of catalyst :(a) Zn2p;(b) Mn2p.

Stability:

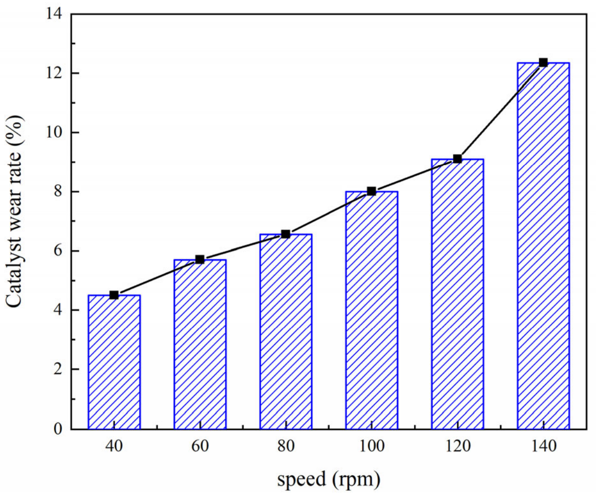

Figure S9. Wear rate of Mn/Zn@PG catalyst.

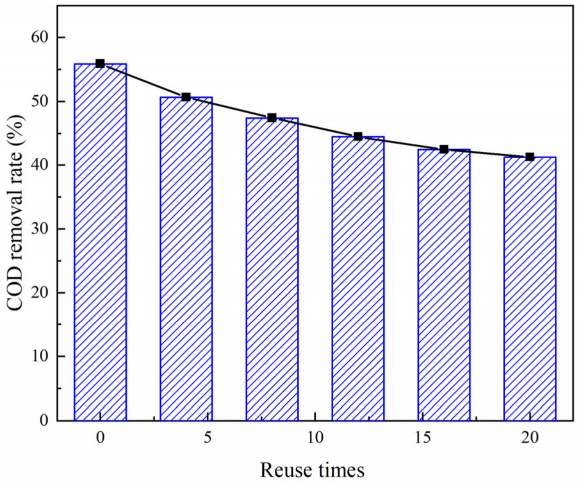

Figure S10. Effect of multiple use on catalyst stability.

TEXT S2 Evaluation model results

The expression of principal components can be determined according to the comprehensive score coefficients:

$$y_i = d_{ij} \cdot a_{ij}^{*}(i = 1,2,\cdots,n, j = 1,2,\cdots,m) \quad \text{(S15)}$$

The expression of principal components:

$$y_1 = 0.084 \cdot x_1 + 0.084 \cdot x_2 + 0.086 \cdot x_3 - 0.056 \cdot x_4 + 0.005 \cdot x_5 + 0.040 \cdot x_6 + 0.010 \cdot x_7 \quad \text{(S16)}$$

$$y_2 = 0.062 \cdot x_1 - 0.030 \cdot x_2 - 0.033 \cdot x_3 + 0.089 \cdot x_4 - 0.041 \cdot x_5 + 0.116 \cdot x_6 + 0.118 \cdot x_7 \quad \text{(S17)}$$

$$y_3 = 0.021 \cdot x_1 + 0.031 \cdot x_2 - 0.005 \cdot x_3 + 0.037 \cdot x_4 + 0.239 \cdot x_5 + 0.001 \cdot x_6 + 0.029 \cdot x_7 \quad \text{(S18)}$$

$$y = 0.534 \cdot y_1 + 0.169 \cdot y_2 - 0.145 \cdot y_3 \quad \text{(S19)}$$

Table S14. Ranking of comprehensive scores.

Operating condition	PC1	PC2	PC3	Comprehensive score	Ranking
1	-0.10	-0.12	-0.02	-0.08	13
2	0.15	-0.01	-0.01	0.08	6
3	0.26	0.12	0.00	0.16	3
4	0.35	0.25	0.00	0.23	2
5	0.38	0.40	-0.02	0.27	1
6	-0.09	0.10	-0.64	-0.12	15
7	0.04	0.04	-0.33	-0.02	11
8	0.13	-0.08	0.33	0.10	5
9	0.02	-0.17	0.63	0.07	8
10	-0.24	-0.48	-0.08	-0.22	17
11	-0.06	-0.32	-0.06	-0.09	14
12	0.05	-0.18	-0.04	-0.01	10
13	0.15	-0.05	0.00	0.07	9
14	0.26	0.08	0.04	0.16	4
15	0.20	-0.13	-0.03	0.08	7
16	-0.17	0.07	0.05	-0.07	12
17	-0.39	0.16	0.08	-0.17	16
18	-0.94	0.32	0.10	-0.43	18
